# Development and evaluation of alginate-based gastroretentive raft-forming systems enabling sustained release of propolis in gastric ulcer treatment

**DOI:** 10.1080/10717544.2026.2616887

**Published:** 2026-01-21

**Authors:** Haniye Takbirgou, Maryam Salami, Gholamreza Askari, Zahra Emam-Djomeh, Raimar Löbenberg, Michael J. Serpe, Neal M. Davies, Wing-Fu Lai

**Affiliations:** a Department of Food Science and Engineering, University College of Agriculture & Natural Resources, University of Tehran, Karaj Campus, Karaj, Iran; b Functional Food Research Core (FFRC), University of Tehran, Tehran, Iran; c Faculty of Pharmacy and Pharmaceutical Sciences, University of Alberta, Edmonton, Canada; d Department of Chemistry, Faculty of Science, University of Alberta, Edmonton, Canada; e School of Food Science and Nutrition, University of Leeds, Leeds, United Kingdom

**Keywords:** Alginate, raft system, reflux disorders, propolis, drug delivery

## Abstract

This study aimed to develop alginate-based raft-forming systems incorporating propolis–whey protein isolate (5%) nanocomplexes to alleviate reflux symptoms and enable the sustained, gastric-specific delivery of propolis for ulcer management. Propolis–protein complexes were prepared at four ratios (2.5:100, 5:100, 7.5:100, and 10:100 w/v) by heating at 85 °C for 5 h at pH 2, producing nanofibrils characterized by thioflavin T fluorescence, intrinsic fluorescence, encapsulation efficiency (up to 78%), and antioxidant activity. The optimal complex was incorporated into alginate rafts at 5%, 10%, and 15% (w/v). Rafts exhibited prolonged floatability (>8 h), increased thickness (from 3.2 ± 0.4 mm to 4.7 ± 0.3 mm with higher propolis loading), enhanced mechanical strength (up to 1.6-fold improvement), and improved reflux resistance. SEM imaging revealed a more compact and uniform porous structure, while FT-IR confirmed molecular interactions between alginate and the propolis–protein complex. *In vitro* release studies in simulated gastric fluid showed suppression of initial burst release, with sustained propolis release over 6–8 h. Overall, alginate rafts containing propolis–protein nanocomplexes demonstrated enhanced structural performance, controlled release behavior, and promising potential for targeted gastric delivery in the management of gastric ulcers.

## 1. Introduction

Oral administration is the most convenient and widely adopted route with high patient compliance for drug delivery, representing approximately 90% of all therapies used in the treatment of gastric diseases (Bhattarai et al. 2010). However, some drawbacks, such as pH, high enzymatic activity, or rapid gastrointestinal transit, can prevent complete drug release from the dosage form and decrease the bioavailability of the delivered drug (Iglesias et al. 2020). One strategy to address these problems is the development of gastroretentive drug delivery systems (GRDDs), which are systems that are retained in the stomach for an extended period and thereby enhance the bioavailability of drugs. This approach can improve the bioavailability of poorly soluble drugs, which is particularly important for drugs with a narrow therapeutic window, as well as controlling the release of bioactive compound at the desired rate for up to several hours in a sustained manner (Tamargo et al. 2015).

Among the main types of GRDDs developed, alginate-based raft-forming systems have attracted much attention because of not only their ability to prevent gastroesophageal reflux disorders but also to promote the sustained release of loaded drugs. Moreover, since physical raft can be retained in the stomach for several hours, raft-forming systems provide longer-lasting relief than traditional antacids. In addition, the absence of complex manufacturing processes and ease of administration for patients are other benefits of raft-forming delivery systems (Prajapati et al. 2013). Raft systems usually contain sodium alginate (as the main gel-forming compound), along with sodium bicarbonate and calcium carbonate (as the main agents used to create carbon dioxide gas). Briefly, upon contact with gastric fluids, alginate forms a cohesive gel, which has the potential to entrap CO_2_ bubbles produced from sodium bicarbonate and calcium carbonate. This process results in a reduction in the density of the system, leading to the formation of a foam layer on top of the stomach contents, like a raft on water (Hanif and Abbas 2018).

Until now, many studies have shown that alginate-based raft formulations are promising candidates for encapsulating therapeutic agents within the matrix to enhance their controlled release at a specific site. In this context, Abbas and Hanif (2018) evaluated the physicochemical properties of an alginate-pectin raft and examined the in vitro release of pantoprazole sodium sesquihydrate from the raft. Their results showed that the raft-forming system successfully delivered the drug in a controlled manner and improved the bioavailability of the medicine. Currently, consumer demand for products containing natural additives has increased due to the side effects of synthetic drugs. For this purpose, the incorporation of flavonoids as antioxidants and preservatives into raft-forming systems has gained considerable interest. Curcumin (Kerdsakundee et al. (2015), quercetin (Bunlung et al. (2021), and lycoside-rich centella extract (Wannasarit et al. (2020) are examples that are incorporated into alginate rafts to control the delivery of dosage forms in the stomach up to several hours by continuously releasing bioactive compounds in a sustained manner.

Propolis has a diverse chemical composition containing a high concentration of flavonoids and phenolic compounds. It is a sticky, resinous, and waxy-like mixture produced by bees from substances collected from beeswax and plant exudates. Propolis has shown a wide range of health properties, such as anti-inflammatory, antimicrobial, wound healing, anticarcinogenic, and antioxidant activities (Busch et al. (2017). Additionally, propolis has an effective potential to be developed as a material for protection against stomach ulcers because it contains various phenolic compounds (Barros et al. (2008), is effective in treating gastrointestinal disorders (Silva et al. (2018; Boeing et al. (2021), and is a good pharmaceutical agent for the inhibition of *Helicobacter pylori* in the stomach (Baltas et al. (2016). Current evidence strongly confirms that propolis ethanolic extracts (PEEs) exert potent gastroprotective effects through antioxidant, anti-inflammatory, antimicrobial, and mucosal-strengthening mechanisms. Numerous in vitro experimental studies have shown that PEE significantly reduces mucosal lesions (Nakamura et al. (2014) and exhibits both anti-inflammatory and antinociceptive activities (de Mendonça et al. (2020). PEE has also been shown to exhibit gastroprotective effects against indomethacin-induced gastric ulcers (Ruiz-Hurtado et al. (2021), as well as ethanol-, indomethacin-, and stress-induced ulcers (de Barros et al. (2007). Moreover, ethanolic extracts of green propolis have been shown to promote both gastroprotective and gastric healing effects associated with enhanced mucin production, increased proliferation of mucosal cells, and restored oxidative balance. Artepillin C, the major component of green propolis extracts, has been identified as a key factor responsible for exerting these gastroprotective effects (Costa et al. (2020). Furthermore, evidence shows that PEE alleviates *H. pylori*-induced injury in gastric epithelial cells by suppressing the inflammatory pathways of nuclear factor-kappa B (NF-κB) and mitogen-activated protein kinases (MAPKs) while enhancing antioxidant responses through the pathway of Nrf2 activation (Song et al. (2020). Despite the promising potential mentioned above, the use of propolis in food or medicinal formulations is limited due to its low water solubility, pungent odor, and bitter taste. The latter might affect drug sensory properties and mouthfeel. In this regard, it has been proven that propolis encapsulation can be a good strategy for hiding its bitter qualities and improving customer acceptability. This method prevents the drug from electrostatic interactions with chemoreceptors by delaying its release into the oral cavity (Bodini et al. (2013).

Over the years, protein nanofibrils have been regarded as promising candidates in drug delivery because they are biocompatible and resistant to heat or a wide range of pH levels. These unique characteristics can protect drugs and bioactive components from degradation when they are exposed to an acidic medium in the stomach (Mohammadian and Madadlou (2018). In this context, Shakoury et al. investigated the possibility of encapsulating propolis extract in the nanoparticles of whey protein (WPN) to enhance the functionality, physicochemical characteristics, and controlled release of propolis in the gastrointestinal tract (Shakoury et al. (2022). In another study, Keskin et al. (2019) encapsulated PEE in sodium alginate beads and evaluated the release behavior of the beads in simulated gastrointestinal conditions (Keskin et al. (2019). However, bead-based encapsulation generally has some inherent limitations that have to be addressed. For example, bead-based systems often have limited gastric retention, which may decrease site-specific therapeutic effects in gastric disorders. In other words, the pH-responsive nature of alginate beads causes them to release more in the intestinal fluid than in the acidic environment of the stomach, making them less effective for targeting stomach ulcers. In contrast, alginate raft systems provide a floating gel layer that can retain bioactive compounds for prolonged periods in the gastric medium, thereby enhancing both their bioavailability and sustained release in the stomach. Therefore, considering both the PEE potential in treating gastrointestinal problems and the dual effects of alginate rafts in reflux disorders and drug delivery, in this work, we took into account that the incorporation of propolis into alginate rafts could be effective for not only treating ulcers but also controlling the release of propolis in the stomach. However, as mentioned above, propolis may affect the sensory characteristics of drug formulations because of its pungent taste and low solubility. To overcome this limitation, we speculated that WPN could be a good candidate to encapsulate propolis. In this context, the purpose of this research was to investigate the possibility of using WPN–propolis in alginate rafts. Moreover, the physicochemical characteristics and release behavior of the resulting system were evaluated to examine its potential as a new gastroretentive drug delivery system.

## 2. Materials and methods

### 2.1. Materials

Sodium alginate was obtained from Sigma-Aldrich (St. Louis, MO, USA), and the inherent viscosity of the 1% solution was approximately 12.8 mPa·s (range: 5 to 40 mPa·s; lot number: MKBT7870V). Sodium bicarbonate and calcium carbonate were purchased from Merck (Darmstadt, Germany). Propolis samples were collected in East Azerbaijan Province, Iran. Whey protein isolate (WPI) was purchased from Merck (Darmstadt, Germany). DPPH (2,2-diphenyl-1-picryalhydrazyl) was purchased from Sigma (Sigma-Aldrich, St. Louis, MO, USA). Pepsin was kindly provided by Bio Basic Company (BIO BASIC, Inc., Ontario, Canada), and double-distilled water was used in the entire study.

### 2.2. Propolis extraction and determination of total phenolic compounds

PEE was obtained using the method previously reported by Busch et al., with some modifications (Busch et al. (2017). At first, the propolis was ground into a powder using a grater. Then, extraction was performed for 24 h with an ethanol/water (80/20, v/v) mixture at room temperature (1:10, w/v) using a constant stirrer. To remove all remaining wax, the ethanolic extract was kept in the freezer overnight and then centrifuged at 8000 × *g* for 15 min. This process was repeated for four days to remove the wax content. Finally, the clear supernatant was evaporated under reduced pressure, lyophilized, and kept at 4 °C for further analysis. The total phenolic compounds were determined using the method reported by Jansen-Alves et al. (2018) and calculated as mg gallic acid equivalent per gram of dried propolis (mg GAE/g).

### 2.3. Preparation of WPN-PEE

To prepare WPN-PEE, the whey protein fibrillation process was performed according to the method described by Zhang et al. with slight modifications (Zhang et al. (2021). First, a solution of whey protein isolate (WPI, 5% w/v) was prepared in distilled water containing 0.002% (w/v) sodium azide to inhibit microbial growth and kept overnight to ensure complete hydration. Afterward, the pH of the WPI solution was adjusted to 2.0 with 8 M HCl. To encapsulate propolis with WPN, lyophilized propolis was first dissolved in ethanol (with a maximum concentration of 0.2% v/v in the solution). Propolis was added to the WPI solution at the ratio of propolis to protein of 2.5:100, 5:100, 7.5:100, and 10:100 (w/w). Then, the solutions were heated for 5 h at 85 °C under constant stirring. Afterward, the solutions were placed in cold water to stop the fibrillation process. Finally, the pH of each solution was adjusted to 7, and the solutions were lyophilized for further experiments. The encapsulated propolis samples were named as follows: WPN-PEE (2.5%), WPN-PEE (5%), WPN-PEE (7.5%), and WPN-PEE (10%). A control sample without propolis was also produced and designated WPN.

### 2.4. Characterization of WPN-PEEs

#### 2.4.1. Determination of WPN-PEEs fibrillar behavior using thioflavin T (Th T)

Briefly, a 10 mM aqueous stock solution of Th T was prepared and filtered through a 0.2 μm filter. Then, 10 μL of each sample was mixed with 490 μL of Th T solution (25 μM). A fluorescence spectrometer (Cary Eclipse, Varian Co, Australia) was used to analyze the fluorescence spectra. The fluorescence signals were recorded at an excitation wavelength of 440 nm and at an emission wavelength of 460–600 nm, respectively (Ansari and Eslami (2020).

#### 2.4.2. Fluorescence spectroscopy

Fluorescence spectroscopy (Cary Eclipse, Varian Co, Australia) was used to analyze the binding mechanism of propolis to WPN. The samples were diluted in PBS (pH = 7) to reach a final protein concentration of 2 mg/mL, according to the method described by Takbirgou et al. (2020). Experiments were conducted using a 1 cm path length fluorescence cuvette and a 5 nm slit width. Emission spectra were individually recorded from 300 to 450 nm with an excitation wavelength of 280 nm for protein and from 370 to 600 nm with an excitation wavelength of 337 nm for propolis.

#### 2.4.3. Encapsulation efficiency (EE)

The EE of propolis was determined according to the method described by Jansen-Alves et al. (2019). Briefly, WPN-PEE samples (50 mg) were mixed with ethanol (10 mL) and centrifuged (4500 × *g*, 15 min, at ambient temperature) to wash non-encapsulated polyphenolic compounds. After phase separation, 1 mL of the supernatant was used to determine the total phenolic compounds (TPCo). The rest of the solution was homogenized and kept in a water bath under stirring for 24 h to release the chemicals of interest from the nanoparticles. Each solution was kept to determine the total phenolic compounds inside the nanoparticles (TPCi). For the determination of the phenolic compounds in the WPN-PEE samples, the Folin‒Ciocalteu method was used. Folin–Ciocalteu reagent (0.2 M) was added to the samples (1 mg/mL) at a ratio of 5:1. Afterward, 2 mL of freshly prepared saturated aqueous sodium carbonate solution was added, the samples were vortexed, and all the samples were incubated for 2 h in the dark. Finally, the absorbance of each sample was determined at 725 nm using a spectrophotometer (SP-UV 500 dB). A standard curve (with a regression coefficient of 0.9964) using gallic acid was also plotted. The EE of the WPN-PEE samples was determined using the following Equation (1):

(1) $$\mathrm{EE}\;\left(\%\right)=\frac{\mathrm{TPCi}-\mathrm{TPC}o}{\mathrm{TPCi}}\times 100.$$

#### 2.4.4. Determination of antioxidant activity

A DPPH radical scavenging assay was used to determine the antioxidant activity of each sample according to the method of Abdullah et al., with some modifications (Abdullah et al. (2019). In summary, 1 mL of the samples was added to 3 mL of 0.1 mM DPPH solution in 96% ethanol at room temperature. The mixtures were incubated for 30 min in the dark. Then, the samples were centrifuged (4000 × g, 10 min), and the absorbance of the supernatant was measured using the UV–Vis spectrophotometer. Equation (2) was used to calculate the antioxidant activity of the samples, where AC and AS were the absorbance of the control and samples at a wavelength of 517 nm, respectively.

(2) $$\mathrm{Ra}\mathrm{dical}\;\mathrm{scavenging}\;\mathrm{activity}\;(\%)=\frac{\mathrm{AC}-\mathrm{AS}}{\mathrm{AS}}\times 100.$$

### 2.5. Preparation of alginate-raft suspension

To obtain the alginate suspension, stock aqueous solutions of sodium alginate, calcium carbonate, and sodium bicarbonate were prepared according to a previously described method (Takbirgou et al. (2024). The amount of each ingredient in the formulations was chosen according to the Gaviscon liquid formula, as listed on the bottle label. In all the samples, alginate, CaCO_3_, and NaHCO_3_ were 5, 1.60, and 2.67 g, respectively. Briefly, alginate powder was dissolved in a portion of distilled water and kept for 24 h to achieve a uniform gel. The gel was stirred, and calcium carbonate was added and mixed well until complete dissolution was reached. Then, sodium bicarbonate was dissolved in distilled water in a separate beaker under constant stirring at ambient temperature and mixed thoroughly with other components. In the case of propolis-incorporated materials, WPN-PEE was added at rates of 5, 10, and 15% (w/v), and mixed well with other ingredients. The final volume of all formulations reached 100 mL with distilled water. The code name of each prepared formulation is given in Table 1.

**Table 1.** The code name for different raft-forming suspensions.

Code	Components of the formulation
ALG	Sodium alginate, calcium carbonate, and sodium bicarbonate
ALG–WP (5%)	Sodium alginate, calcium carbonate, sodium bicarbonate, and WPN-PEE (5%)
ALG–WP (10%)	Sodium alginate, calcium carbonate, sodium bicarbonate, and WPN-PEE (10%)
ALG–WP (15%)	Sodium alginate, calcium carbonate, sodium bicarbonate, and WPN-PEE (15%)

Abbreviations: ALG refers to the alginate-based raft formulation (control), while ALG–WP (5%), ALG–WP (10%), and ALG–WP (15%) indicate alginate-based raft-forming formulations containing 5%, 10%, and 15% (w/v) whey protein nanofibril–propolis extract, respectively.

### 2.6. Physicochemical characterization of raft-forming liquid formulations

#### 2.6.1. Viscosity measurement

The viscosity of each sample was determined using a rotational viscometer (Physica MCR 301, Anton Paar, Austria) at 25 °C with a cylindrical LV spindle (No. 18). For each test, approximately 20 mL of suspension was poured into the vessel, and the shear rate was set at 20 rpm.

#### 2.6.2. Floating behavior of rafts

150 mL of the medium (0.1 M HCl) was poured into a 250 mL beaker, and the temperature was set at 37 °C. Then, 20 mL of the suspension was added to the medium. The floating lag time was measured when the formulation first appeared on the surface of the acidic medium. The total floating time was determined when the formulation consistently remained floating on the acidic medium (Moazen et al. (2022).

#### 2.6.3. Raft volume

The raft volume was calculated according to the method previously reported by Abbas et al. (2017). Briefly, a 250 mL beaker was weighed (W_B_) and filled with 150 mL of 0.1 M HCl. Then, 20 mL of each sample was poured into the weighed beaker and allowed to develop the raft for 30 min. At this stage, the height reached by the top of each raft was marked on the outside of the beaker, and the total weight of the beaker and its contents was recorded (W_T_). Then, the gel was carefully collected with a small spoon and weighed (W_R_). Finally, the HCl was removed, and the beaker was refilled with water up to the marked raft height and weighed again (W_W_).

The raft volume was calculated using Equation (3):

(3) $$\mathrm{Raft}\;\mathrm{volume}=({\text{W}}_{W}-{\text{W}}_{B})-({\text{W}}_{T}-{\text{W}}_{B}-{\text{W}}_{R}).$$

#### 2.6.4. Raft thickness

For thickness measurements, rafts were formed in a 250 mL beaker by adding 20 mL of suspension to 150 mL of 0.1 M HCl, which was pre-equilibrated at 37 °C. Measurements were performed at three places around the beaker by a digital Vernier caliper (Shandong, China) and expressed as a mean value (Hanif and Abbas (2018).

#### 2.6.5. Raft strength

A texture analyzer (CT3™, Brookfield Engineering Inc., USA) equipped with an L-shaped steel wire with a diameter of 1.2 mm was used to determine the strength of the rafts (Hampson et al. (2005). Briefly, an L-shaped wire was fixed in the center of the beaker, which was pre-filled with 150 mL of 0.1 M HCl. The alginate suspension (20 mL) was then poured into the beaker, and the raft was allowed to form around the wire while the temperature was set at 37 °C during analysis. After 30 min, the wire probe was elevated throughout the raft at a rate of 5 mm/s, and the raft strength was recorded as the force required to pull the wire up through the raft.

#### 2.6.6. Raft resilience

Rafts were formed in a 250-mL glass jar. Then, the jar was placed in a tumble mixer (Turbula T2C, W.A. Bachofen AG, Basel), which was set at 20 rpm to simulate gastric agitation. Rafts were observed visually at 5, 20, 40, and 60 min or until the raft was no longer detectable. Raft resilience was defined as the last time point at which the raft began to disintegrate.

#### 2.6.7. Reflux resistance

A texture analyzer (CT3™, Brookfield Engineering Inc., USA) equipped with a TA 25/1000 plunger was used to determine the reflux resistance according to the method described previously by Takbirgou et al. (2024). Briefly, to simulate conditions in the stomach, cylinders with 10-, 15- and 20-mm diameter orifices at their bottoms were designed. After covering the orifice, each cylinder was filled with 150 mL of HCl (0.1 M, 37 °C), followed by adding 20 mL of the suspension to develop rafts for 30 min. Then, the orifice was opened to allow the liquid to flow out. Afterward, the cylinder was placed on the platform of the texture analyzer, and the TA 25/1000 plunger was descended at a rate of 5 mm/s for 15 mm through the raft. The reflux resistance was measured as the force needed to push the raft through the orifice.

#### 2.6.8. Morphology analysis

The morphology of ALG and ALG–WP (15%) was examined using scanning electron microscopy (SEM, Madell Technology Corporation KYKY-EM 3200, USA) at 500× magnification and an acceleration voltage of 15 kV. Briefly, small amounts of each sample (rafts of ALG and ALG–WP (15%)) were collected and lyophilized. Then, the samples were mounted on aluminum stubs and sputter-coated with gold before imaging.

#### 2.6.9. Fourier transform infrared spectroscopy (FT-IR)

FT-IR spectroscopy was used to study the molecular properties and interactions of propolis, WPN-PEE, ALG, and ALG–WP (15%) using an FT-IR spectrometer (Billerica, Massachusetts, USA). The samples were lyophilized and pressed into potassium bromide discs. Measurements were recorded in transmittance mode in the range of 4000 and 500 cm^−1^.

#### 2.6.10. In-vitro propolis release

The release rate of propolis from alginate raft-forming systems was investigated using the method of Bunlung et al. (2021), with some changes. A total of 900 mL of 0.1 N hydrochloric acid with a final pH value of 1.2, comprising pepsin at a ratio of 1:250 enzyme to WPN (w/w), was used as the release medium. The temperature was maintained at 37 °C with continuous shaking at a rate of 50 rpm. Aliquots of each release medium (1 mL) were withdrawn at specific time intervals (60, 120, 180, 240, 300, and 360 min), and the same volume was returned to the system to maintain the sink condition. The absorbance of the propolis in the withdrawn samples was measured at a wavelength of 337 nm using a UV spectrophotometer. The test was repeated three times for each formulation, and the data were reported as the mean ± SD. A plot of the cumulative release of propolis against time was constructed to illustrate propolis release profiles. A propolis standard curve was used to calculate the concentration of the released propolis, which was measured spectrophotometrically at 337 nm.

#### 2.6.11. Mechanism of propolis release

The release data were fitted to various kinetic models (zero-order, first-order, Higuchi, and Hixson–Crowell) to analyze the kinetics of drug release. To determine the mechanism of drug release, the first 60% of the drug release data were fitted to the Korsmeyer–Peppas model using Equation (4):

(4) $$\frac{M_{t}}{M_{\infty }}=kt^{n},$$

where *M_t_*/*M*_∞_ designates the fraction of drug released at time *t*, *n* is the diffusional exponent indicative of the drug release mechanism, and *k* is the kinetic constant.

### 2.7. Statistical analysis

The significant differences among treatments were evaluated by one-way ANOVA using Duncan's test (*p* ≤ 0.05) with SPSS software (version 25.0, SPSS, Inc., Chicago, IL, USA).

## 3. Result and discussion

### 3.1. Th T assay

**Figure 1.**
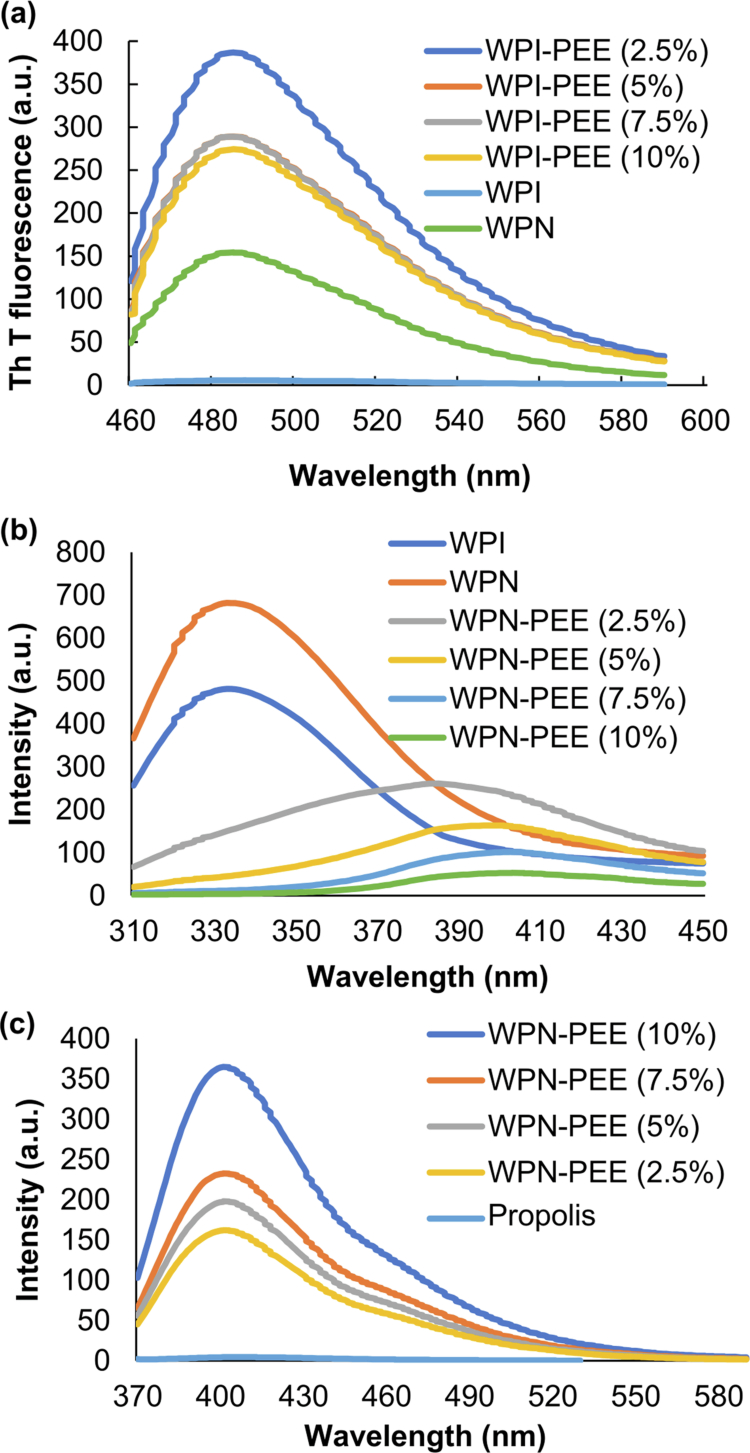
(a) Th T fluorescence emission spectra of WPI and WPN before and after incorporation of propolis upon excitation at 440 nm. (b and c) Fluorescence emission spectra of WPI and WPN before and after incorporation of propolis upon excitation at 280 nm (b) and those of free propolis and WPN complexes with propolis at 337 nm (c).

Th T is a specific dye, commonly used to detect the presence of amyloid fibrillar aggregates, which has a specific binding affinity to β-sheet structures and produces strong fluorescence (Farrokhi et al. (2018). Figure 1a represents the results of Th T fluorescence. Compared with WPI as the control, which showed almost no fluorescence intensity, WPN showed increased Th T fluorescence intensity, indicating that fibrils were formed during the fibrillation process (Serfert et al. (2014). The obtained result also showed that propolis caused a further increase in amyloid formation, indicating a more pronounced α-helical-to-β-sheet structural transition or an increase in fibrillar structure formation. This observation showed that propolis incorporation might affect the fibril growth mechanism or their mature structure at low concentrations. However, from WPN-PEE (2.5%) to WPN-PEE (10%), the growth rate of Th T fluorescence decreased in a dose-dependent manner as a function of the propolis concentration, causing quenching of the fluorescence signal. This phenomenon suggested that the propolis component in WPN-PEE (10%) likely bound more effectively to unfolded WPNs than the other samples. It may be populated between aggregated layers of WPN, leading to stronger inhibition. This is in agreement with other studies, which have shown that propolis can suppress the level of amyloid cross-β-structure of bovine insulin (Ramezani et al. (2021) and glycated human hemoglobin (Kazemi et al. (2019).

### 3.2. Fluorescence quenching and propolis fluorescence

The fluorescence quenching spectra of WPN before and after the incorporation of propolis, upon excitation at 280 nm, were investigated via fluorescence spectroscopy. As shown in Figure 1b, the fluorescence intensity was higher for WPN than WPI because more hydrophobic patches were exposed during heating, resulting in a larger surface area of WPN during the fibrillation process (Mohammadian et al. (2019). After the incorporation of propolis, the intensity of fluorescence decreased, indicating hydrophobic interactions between propolis and tryptophan residues in WPN. Additionally, in samples with higher propolis concentrations, the intensity was lower. This finding is consistent with our Th T fluorescence results, confirming higher loading efficiency. These observations agree with the results of Shakoury et al. (2022), who reported a decreasing trend in fluorescence intensity with the addition of propolis to WPN. Moreover, a significant redshift occurred. This means that tryptophan and tyrosine residues were exposed to a more polar environment, suggesting protein unfolding and strong association of WPN with propolis (Aliyari et al. (2021).

Figure 1c shows fluorescence excitation at 337 nm. In the absence of WPN, propolis showed a low-intensity peak at 405 nm, but in the presence of WPN, the intensity significantly increased. This increase in fluorescence intensity with a slight shift in the emission maximum from longer to shorter wavelength (401 nm) indicates that the movement of propolis from a polar to a less polar environment results from the binding of propolis to the hydrophobic patches of WPN. The data reported here for WPN-PEE are consistent with previous observations of Mohammadian et al. (2019) and Hu et al. (2020), who loaded curcumin into WPN and WPN–chitosan complexes, respectively. They reported that WPN, alone or in complex with chitosan, can be a promising vehicle for curcumin to improve its functional properties.

### 3.3. Encapsulation efficiency

**Figure 2.**
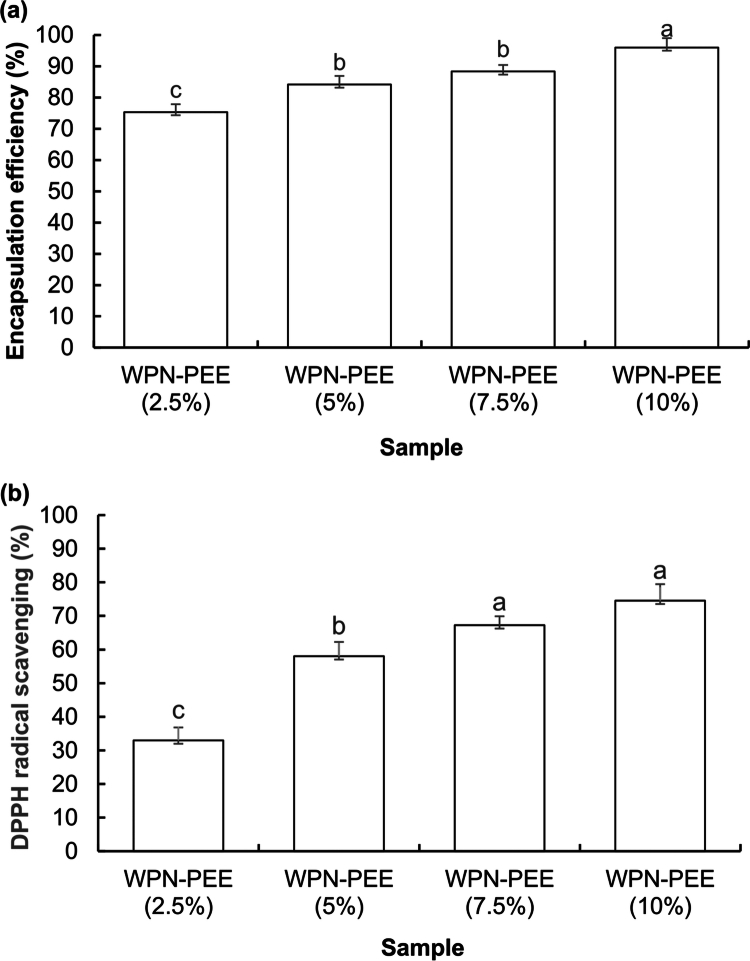
(a) Encapsulation efficiency of WPN complexes with propolis (2.5, 5, 7.5, and 10%). (b) Antioxidant activity of WPN complexes with propolis (2.5, 5, 7.5, and 10%). Different letters indicate significant differences among samples (*p* ≤ 0.05) according to Duncan's test (*n* = 3).

The EE of the samples was evaluated to assess the effectiveness of WPN as a carrier for propolis. Initially, the total phenolic compounds were quantified and recorded (410 mg GAE/g dried material). As shown in Figure 2a, the EE increased with higher propolis loading. Relatively high EE was obtained for all samples (from 75 to 96%), indicating that heating during the fibrillation process can not only affect the degradation of propolis polyphenols on a large scale but also increase the loading efficiency, even at high concentrations of propolis, partly due to an increase in the accessibility of hydrophobic patches for propolis. Jansen-Alves et al. also reported that the EE was greater than 70% for propolis encapsulated by spray drying with the help of rice, pea, soybean, and ovalbumin proteins as wall materials (Jansen-Alves et al. (2018).

### 3.4. Antioxidant activity

In recent years, several studies have been performed to evaluate the antioxidant capacity of natural products (Na et al. (2011). Propolis, which is composed of different polyphenols, has been reported to possess high values of antioxidant activity (Kurek-Górecka et al. (2013; Martinello and Mutinelli (2021 ). Figure 2b illustrated the results of the antioxidant activity (DPPH assay) of encapsulated propolis in WPN. The difference in the antioxidant activity of the samples was due to the difference in the proportion of propolis. The highest antioxidant activity was observed in WPN-PEE (10%), which had the most propolis content. Additionally, the results obtained showed that the heating process was not deleterious for the propolis responsible for the antioxidant activity, as it was high. Considering the abovementioned experiments, WPN-PEE (10%) was used as the best sample for the rest of the experiments. In other words, WPN-PEE (10%) was added to alginate raft suspensions in portions of 5, 10, and 15% (w/v), which were named according to the code mentioned in Table 1.

### 3.5. Viscosity measurement of alginate raft suspensions

A successful liquid pharmaceutical formulation should possess optimum viscosity to allow easy pouring when consumed. The viscosity values of the raft-forming liquid formulations are presented in Table 2. All the solutions exhibited pseudoplastic flow or shear-thinning behavior, which is a desirable factor in pharmaceutical formulations to decrease the sedimentation volume during preservation. The range of viscosity was between 1338 and 4790 mPa·s. The lowest viscosity was observed for ALG (1338 mPa·s), which contained no propolis, showing that WPN-PEE exerted a determining effect on viscosity (*p* < 0.05). A previous study conducted by Takbirgou et al. (2024) reported that adding carbomer and aloe vera powder to raft formulations caused a significant increase in viscosity. In our study, all alginate suspensions had a suitable viscosity except for ALG–WP (15%), which could hardly be poured into a spoon (4790 mPa·s). This behavior can be attributed to the high concentration of WPN-PEE, which increased frictional forces and hence resistance to flow, resulting in an increase in viscosity.

**Table 2.** Results of viscosity, FLT, TFT, and raft volume of formulations.

Formulation	Viscosity (mPa·s)	FLT(s)	TFT(h)	Raft volume (mL) ± SD
ALG	1338	125	>4	56.89 ± 2.31^c^
ALG–WP (5%)	2298	60	>8	62.56 ± 0.58^b^
ALG–WP (10%)	3446	35	>8	64.41 ± 1.15^b^
ALG–WP (15%)	4790	20	>8	71.39 ± 1.86^a^

The values for raft volume are mean ± standard deviation from triplicate determinations. Different superscript letters in the same column represent significant differences in means (*p* < 0.05). Abbreviations: FLT, floating lag time; TFT, total floating time.

### 3.6. Floating lag time and total floating time

**Figure 3.**
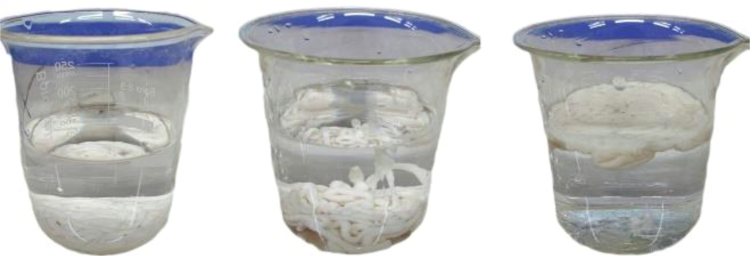
Images of the behavior of the alginate suspension (ALG–WP (15%)) in an acidic medium. The picture showed how the raft developed when exposed to 150 mL of HCl (0.1 M, 37 °C) at the initial moment (left image), after 5 s (middle image), and 20 s (right image).

The raft formation mechanism is based on three steps: (1) alginate conversion to alginic acid when exposed to the acidic condition of the stomach, (2) the discharge of divalent calcium ions present in calcium carbonate, and (3) the reaction of alginic acid with calcium ions to form the raft system. Additionally, in an acidic medium, sodium bicarbonate produces CO_2_, which is entrapped in the gel and finally forms a barrier on top of the stomach contents due to the buoyancy of the matrix (Figure 3). To achieve this purpose, the density of the raft, formed from liquid formulations, should be much lower than the density of the gastric medium. During these steps, the time that the suspension takes to move from the bottom to the medium surface is referred to as the floating lag time (FLT), and the time that the gel persists on the medium surface is referred to as the total floating time (TFT). The results of FLT and TFT are presented in Table 2. As shown, in all formulations containing encapsulated propolis, the FLT was lower than 1 min, demonstrating that WPN-PEE at this range did not lead to formulations with higher density than gastric fluids, and propolis loading had no effect on the floating ability. Similarly, the TFT of the prepared suspensions incorporated with WPN-PEE were more than approximately 8 h, which may be due to the cohesive and integrated structure of the raft resulting from WPN-PEE.

### 3.7. Raft volume and thickness

The raft volume was associated with CO_2_ formation as a result of the dissociation of sodium bicarbonate and calcium carbonate at low pH values. In Table 2, the results of the raft volume are reported. ALG showed the lowest raft volume, approximately 57 mL, which may be attributed to its lower viscosity. In ALG–WP (5%) and ALG–WP (10%), there was no significant difference in raft volume. However, ALG–WP (15%) showed the highest volume, which may be attributed to its rigid structure and the raft's potential to retain CO_2_ gas for a longer time within the gel. The results of raft thickness are reported in Table 3, ranging from 25.33 ± 0.50 to 29.41 ± 0.38 mm. ALG showed the lowest thickness because it had a smaller amount of polymers in its structure. Our findings were in agreement with Abbas and Hanif (2018), who reported that raft thickness was related to the concentration of polymers in the raft.

**Table 3.** Results of the thickness, strength, and resilience of each raft formulation.

Formulation	Raft thickness (mm) ± SD	Raft strength (g)	Raft resilience (min)
ALG	25.33 ± 0.50^d^	9.6	~240
ALG–WP (5%)	26.88 ± 0.46^c^	10.69	~420
ALG–WP (10%)	27.83 ± 0.35^b^	11.54	>420
ALG–WP (15%)	29.41 ± 0.38^a^	12.41	>420

The values for raft thickness are mean ± standard deviation from triplicate determinations. Different superscript letters in the same column represent significant differences in means (*p* < 0.05).

### 3.8. Raft strength

Naturally, alginate sol can be formed through van der Waals attractions at ambient temperature when solubilized in water. In the presence of either divalent or monovalent metal ions, alginate undergoes gelation to form 3-D egg-box structures, leading to the formation of a strong network. In this context, raft strength is one of the most important features that should be evaluated. Different factors affect raft strength. Examples of these factors include the concentrations of ingredients (alginate, calcium carbonate, and sodium bicarbonate), the molecular weight of alginate, sequences of mannuronic (M) and guluronic acid (G) residues, and the ratio of M to G blocks in alginate (Johnson et al. (1997; Donati et al. (2005).

Various methods have been used to measure raft strength (Prajapati et al. (2012; Abbas et al. (2017; Hanif and Abbas (2018). In this study, the method previously described by Hampton and coworkers was adopted to determine the raft strength of the formulations (Hampson et al. (2005). As shown in Table 3, the raft strength of all formulations tested in the present study was approximately 10–12 g and was within the range reported for commercial formulations (4–16 g) (Hampson et al. (2005). The maximum raft strength was observed in suspensions containing WPN-PEE, indicating that the presence of WPN-PEE did not have a negative effect on raft strength.

### 3.9. Raft resilience

Raft resistance to breakup under simulated peristaltic movements in the stomach is referred to as raft resilience. It has been shown that there is a direct correlation between raft resilience and the alginate concentration, raft strength, and dose of the drug used. Hanif and Abbas reported that stronger alginate rafts disintegrated more slowly when exposed to simulated gastric motility conditions (Hanif and Abbas (2018). The results of raft resilience in our experiment are presented in Table 3. It is clear that raft resilience was enhanced in the case of the products with WPN-PEE (approximately 420 min or higher), as expected, due to their higher raft strength compared with ALG, which had no WPN-PEE.

### 3.10. Reflux resistance

Resistance to reflux is a factor indicating the ability of the raft to prevent reflux. The reflux resistance values ranged from 730 to 901 g, 970 to 1224 g, and 1936 to 2706 g through orifices of 20 mm, 15 mm, and 10 mm diameter, respectively, as shown in Table 4. Our results indicated that all the formulations were strong enough to be resistant to reflux. Moreover, a significant difference was observed between reflux resistance at different diameters, which was consistent with previous reports (Moazen et al. (2022; Takbirgou et al. (2024).

**Table 4.** Reflux resistance through 20-, 15-, and 10-mm diameter orifices measured using a texture analyzer.

Formulation	Reflux resistance (g)		
	20-mm diameter	15-mm diameter	10-mm diameter
ALG	730	970	1936
ALG–WP (5%)	763	1122	2182
ALG–WP (10%)	849	1191	2529
ALG–WP (15%)	901	1224	2706

### 3.11. Morphological analysis

**Figure 4.**
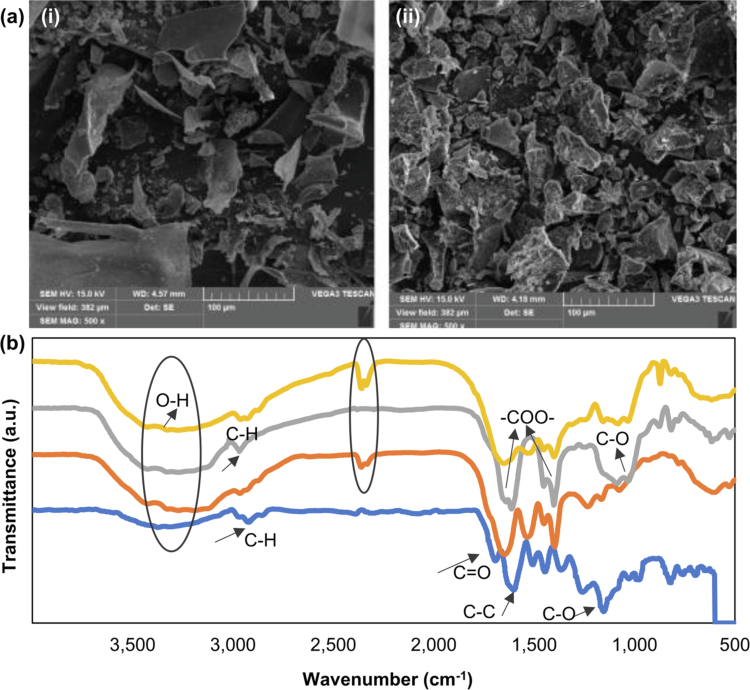
(a) SEM images of dried rafts of (i) ALG and (ii) ALG–WP (15%) at 500× magnification. Scale bar = 100 µm. (b) FT-IR profiles of propolis, WPN-PEE, and alginate rafts before and after incorporating 15% WPN-PEE, shown as blue, orange, gray, and yellow lines, respectively.

SEM images (500× magnification, scale bar = 100 μm) of dried rafts of ALG and ALG–WP (15%) are shown in Figure 4a. The dried rafts of both samples were found to exhibit irregular surface structures with some pores, which can be attributed to CO_2_ gas entrapped in the raft. The ALG sample exhibited large, plate-like fragments with sharp edges. The particles showed loosely fractured lines with relatively smooth surfaces, suggesting a less compact structure. This characteristic results from weaker interactions among the matrixes, which may correspond to the lower surface area as well as the reduced uniformity in the dispersion. In contrast, the ALG–WP (15%) sample revealed a more compact morphology with smaller and more uniformly distributed particles. The increased surface roughness due to the presence of WPN-PEE in the raft showed enhanced particle cohesion and greater structural integrity. Additionally, a more porous structure demonstrated a higher surface area, suggesting better interaction with media and potentially more controlled release behavior. These observations were in agreement with the results of raft strength, showing that WPN-PEE incorporated inside the raft formed a coherent layer, which could be considered an important characteristic of alginate rafts.

### 3.12. FT-IR analysis

FT-IR spectroscopy was utilized to identify the interactions between propolis, WPN-PEE, ALG, and ALG–WP (15%) samples since each functional group absorbs radiation at a characteristic frequency of the infrared spectrum. As shown in Figure 4b, several peaks observed in the propolis spectrum indicated diverse functional groups in its structure. The absorption broad band around 3200 cm^−1^ could be associated with the OH stretching vibration of the hydroxyl groups available on phenolic compounds, and the small peak at 2915.08 cm^−1^ could be related to the stretching vibration of C‒H bonds (Correa-Pacheco et al. (2019). The signals at 1600.64 cm^−1^ and 1448.35 cm^−1^, which are typical of aromatic systems, corresponded to the stretching vibrations of C─C and C─O of flavonoids (Villalobos et al. (2017). The region at 1365 and 1257 cm^−1^ was related to the CH_3_ symmetrical vibrations, and the bands between 1153 and 1029 cm^−1^ corresponded to C─O bond vibrations of polyphenols (Irigoiti et al. (2021).

Previous studies have shown that the encapsulation process does not affect WPN's secondary structure since the position of the WPN characteristic peaks does not change. However, when a hydrophobic compound is encapsulated in a protein, the height of the protein peak may increase due to the formation of hydrogen bonds and hydrophobic interactions (Le Bourvellec and Renard (2012; Xue et al. (2018; Chang et al. (2020). In our experiment, when propolis was incorporated into WPN, some of the peaks related to the propolis disappeared, probably due to limited stretching and bending vibrations in propolis when bound to WPN (Xue et al. (2018; Mohammadian et al. (2019; Shakoury et al. (2022). In the case of WPN-PEE, the observed peak around 3200 cm^−1^ could be related to OH stretching vibrations. The band that appeared at 2362 cm^−1^ might be attributed to the disruption of some bonds in propolis, followed by the formation of new bonds with WPN throughout the fibrillation process, causing a new peak in WPN-PEE. The band at 1647 cm^−1^ was due to the C═O stretching vibrations of amide I. The band at 1534 cm^−1^ was assigned to N─H bending and C─N stretching vibrations of amide II, reflecting the conformational structure of whey protein nanofibrils interacting with the propolis extract. Additionally, the amide III band around 1400–1200 cm^−1^ was associated with N─H bending and C─H stretching vibrations (Mohammadian and Madadlou (2016). Overall, the broadening of O─H and C─H regions compared to pure propolis suggested potential hydrogen bonding and noncovalent interactions.

In the case of the ALG raft, bands around 3500 and 2960 cm^−1^ were observed, providing information about hydroxyl and methylene stretching vibrations, respectively. The peaks around 1600 and 1400 cm^−1^ were detected and attributed to the asymmetric and symmetric stretching vibrations of ─COO. In the fingerprint region, the bands at 1027 and 815 cm^−1^ were attributed to C─O bond stretching and C─C skeletal vibrations, respectively (Dettmar et al. (2018). Additionally, the ALG formulation showed a peak at 873 cm^−1^, attributed to the CO_3_^2−^ group, suggesting that carbonate was entrapped in the isolated rafts (Jakaria et al. (2015).

In the case of ALG–WP (15%), all the characteristic peaks associated with the rafts were observed. Additionally, some characteristic peaks associated with WPN-PEE were found (e.g. the peak at 2358 cm^−1^). However, the ALG–WP (15%) formulation demonstrated shifts and intensity changes in functional regions associated with both alginate and WPN-PEE components, suggesting electrostatic interactions or partial complexation between alginate and protein. Additionally, the partial disappearance of some characteristic propolis peaks in ALG–WP (15%) indicated specific noncovalent molecular interactions, including hydrogen bonding of phenolic groups with carboxylate and amide groups in alginate and WPN. Furthermore, aromatic residues in proteins can be engaged in π–π interactions. Therefore, considering the presence of aromatic moieties in both propolis and proteins in our system, it can be inferred that noncovalent π–π stacking interactions may contribute to raft stabilization. Overall, these findings suggested that WPN-PEE was compatible with the alginate raft matrix and that the interactions were likely noncovalent.

### 3.13. Release behavior

**Figure 5.**
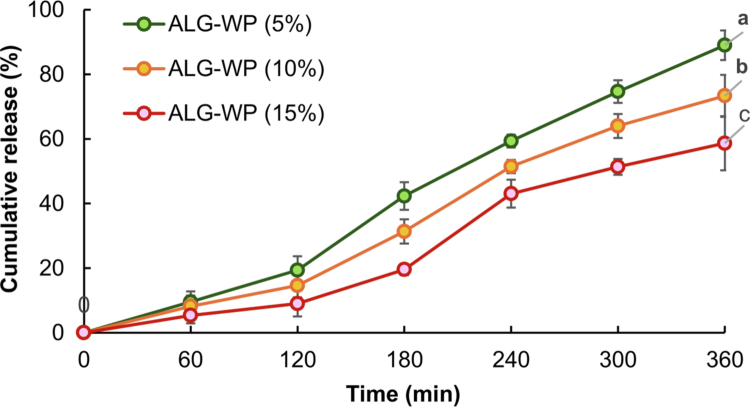
Release profiles of propolis from alginate raft-forming formulations at pH 1.2. The data are presented as mean ± SD (*n* = 3). Different letters indicate significant differences (*p* < 0.05) among different formulations at 360 min.

Regardless of the health benefits of propolis, its bioavailability in the human body is limited, partly because of its poor water solubility. The encapsulation of propolis can potentially enhance the bioavailability by improving the solubility. The rafts formed by our gastroretentive delivery systems are capable of floating on stomach contents for several hours, leading to an increase in the gastric residence time for sustained propolis release. Since the main goal of this study was to develop alginate rafts for the treatment of reflux and gastric ulcer–related disorders, the target site for extract release was only the gastric environment in acidic medium at 37 °C for up to 360 min. The thickness of the raft and the carbon dioxide entrapped in it, resulting from polymer concentration and sodium bicarbonate, create an obstruction to drug release from the raft (Abbas et al. (2017). The results of propolis release from raft-forming formulations in our experiment are presented in Figure 5. In all formulations, the propolis release rate was low before 2 h, demonstrating the effectiveness of the raft in preventing burst release of propolis when exposed to stomach acid. After this time, the release of propolis increased, which was attributed to the release of calcium ions into the medium that weakened the raft structure and caused more propolis to be released into the simulated stomach environment. On the other hand, in formulations with higher WPN-PEE concentrations, the drug took longer to diffuse from the raft because of the higher density and thicker structure of the raft.

The *r*^2^ values ​​obtained after fitting the release data to various kinetic models are shown in Table 5. The release profiles of the formulations fit the zero-order model best. Therefore, the rate of drug release appears to be constant during the experiment, being independent of the concentration of drug remaining in the delivery system. This is highly favorable in a practical sense because, compared with delivery systems exhibiting other patterns of release kinetics, zero-order systems can more effectively maintain drug concentrations within the therapeutic window for an extended period of time, enabling a reduction in the dosing frequency and the occurrence of adverse effects. Furthermore, to determine the release mechanism of the formulations, 60% of the release data were fitted to the Korsmeyer‒Peppas equation. The *n* values ​​ranged from 1.43 to 2.34, indicating that the formulations followed the mechanism of non-Fickian super case II transport. This implies that the process of drug release from our formulations is associated, at least in part, with the stress and state transition in alginate molecules that swell in water or biological fluids. In other words, the drug release process is mediated by the macromolecular relaxation of the polymeric chains (Korsmeyer et al. (1983).

**Table 5.** Release kinetic parameters of different formulations.

	Zero order		First order		Higuchi		Hixson–Crowell		Korsmeyer–Peppas	
	*K*_0_	*r* ^2^	*K*_1_	*r* ^2^	*K*_H_	*r* ^2^	*K*_HC_	*r* ^2^	*n*	*r* ^2^
ALG–WP (5%)	0.239	0.9817	0.004	0.9036	3.739	0.8131	0.001	0.9345	1.434	0.9928
ALG–WP (10%)	0.206	0.9668	0.003	0.8910	3.203	0.7737	0.001	0.9184	1.649	0.9927
ALG–WP (15%)	0.174	0.9159	0.002	0.8459	2.662	0.6985	0.001	0.8701	2.342	0.9837

## 4. Conclusion

In this study, alginate-based raft-forming systems were developed for the treatment of reflux disorders and the controlled release of propolis. Propolis, despite its known therapeutic benefits, presents formulation challenges because of its pungent odor, bitter taste, and low water solubility. To address these issues, an encapsulation technique was employed to mask its strong sensory properties and enhance its solubility in aqueous media. Among the formulations tested, ALG–WP (15%) demonstrated the most favorable functional properties of the raft system and effectively controlled propolis release under gastric conditions. However, increasing the propolis concentration led to higher viscosity and a more pronounced odor in the formulation—characteristics that may be undesirable from a consumer perspective. In conclusion, while the raft-forming system itself exhibits anti-reflux activity, it also serves as a promising delivery vehicle for the targeted release of anti-ulcer propolis in the stomach.

## Data Availability

All data generated or analyzed during this study are included in this published article.
